# Clinical Characteristics and Aggression in Unipolar and Bipolar Course of Affective Disorders

**DOI:** 10.1192/j.eurpsy.2024.894

**Published:** 2024-08-27

**Authors:** L. Smirnova, O. Roshchina, A. Seregin

**Affiliations:** ^1^ Laboratory of Molecular Genetics and Biochemistry; ^2^Affective States Department, Mental Health Research Institute, Tomsk National Research Medical Center of the Russian Academy of Sciences, Tomsk, Russian Federation

## Abstract

**Introduction:**

The diagnosis and treatment of depression are complex due to its diverse forms. Recent focus in clinical practice has been on identifying markers for mono- and bipolar depression, as early diagnosis significantly impacts treatment.

**Objectives:**

To identify clinical characteristics of unipolar and bipolar depressive disorders and assess their correlation with aggression levels in patients.

**Methods:**

We studied patients at the Mental Health Research Institute of Tomsk NRMC: ICD-10 codes: Bipolar Affective Disorder (BD) (n=28), Recurrent Depressive Disorder (RDD) (n=33). Patients with BD were older (49 (33; 52) years) than those RDD (40 (31; 51) years) (p=0.018). The current depressive episode duration was shorter for BD (3 (2; 7) months) compared to RDD (5 (2; 12) months) (p=0.018). Gender distribution was comparable (p=0.568). We measured clinical symptoms (depression, anxiety, anhedonia) using psychometric tools (HAM-D, HAM-A, SHAPS) at admission and after 3 weeks of therapy. Aggression was assessed with the Buss-Durkee Hostility Inventory (BDHI) at admission.

**Results:**

Patients with RDD demonstrated a higher severity of depressive symptoms upon admission (Table 1).

**Table 1.** Clinical Characteristics of Unipolar and Bipolar Depression Course

Severity of Symptoms	Bipolar Depression	Unipolar Depression	р (U-test)
HAM-D on admission	19 (15.5; 24)	22 (18; 26)	**0.044**
HAM-D after 3 weeks	4 (2; 6)	4 (3; 7.75)	0.219
HAM-A on admission	16 (12; 25)	19.5 (13; 26.75)	0.098
HAM-A after 3 weeks	3 (2; 6.5)	4 (3; 7.75)	0.219
SHAPS on admission	5 (1.25; 9)	3 (0; 10)	0.7
SHAPS after 3 weeks	1 (0; 4)	1 (0; 3)	0.44

The severity of some aggressive patterns was higher in patients with bipolar disorder (Table 2).

**Table 2.** The severity of aggressiveness in unipolar and bipolar depression.

BDHI subscale	Bipolar Depression	Unipolar Depression	р (U-test)
Aggressiveness index	19 (13; 24)	18.5 (12; 24)	0.745
Hostility index	9 (7; 13.75)	9 (7; 11)	0.139
Assault Hostility	4 (2; 6)	4 (2; 6)	0.618
Indirect Hostility	5 (5; 6)	4 (4; 6)	**0.015**
Irritability	6 (4; 8)	5 (3; 7)	0.081
Negativism	2 (1; 4)	2 (1; 4)	0.262
Resentment	5 (4; 6)	5 (3; 6)	0.113
Verbal Hostility	7 (6; 8)	6 (5; 8)	**0.008**

As a result of the study, no statistically significant correlations were found (p>0.05, Spearman’s test).

**Conclusions:**

The conducted research did not yield convincing data that would allow us to make judgments about specific clinical patterns in the course of unipolar and bipolar depression. Thus, the problem of searching for unique biological markers of the courses of affective disorders remains relevant. Support by the Russian Science Foundation grant No. 23-75-00023.

**Disclosure of Interest:**

None Declared

